# Differences in health state valuation for small, low-risk thyroid cancer between general population and cancer survivors: a cross-sectional analysis

**DOI:** 10.1007/s11136-025-04033-7

**Published:** 2025-08-23

**Authors:** Kendyl Carlisle, Rebecca Kowalski, Aprill N. Park, Salome Ricci, Kai Sun, Carrie Cunningham, Julia F. Slejko, C. Daniel Mullins, Yinin Hu

**Affiliations:** 1 Department of Surgery, University of Maryland School of Medicine, Baltimore, MD USA; 2 University of Maryland School of Medicine, Baltimore, MD USA; 3 Department of Practice, Sciences, and Health Outcomes Research, University of Maryland School of Pharmacy, Baltimore, MD USA; 4 Department of Epidemiology and Public Health, University of Maryland Baltimore, Baltimore, MD USA; 5 Department of Surgery, Mass General Brigham, Boston, MA USA

**Keywords:** Thyroid cancer, Value, Utility, Time trade-off, Papillary, Microcarcinoma

## Abstract

**Purpose:**

A low-risk cancer characterized by slow growth and excellent prognosis, papillary thyroid microcarcinoma (PTMC) is increasingly managed with less invasive alternatives to surgical resection, including active surveillance and radiofrequency ablation. To inform shared decision-making and comparative-effectiveness models, treatment preferences/quality of life quantified by health utilities must be derived for PTMC. However, there is ambiguity regarding the population from which these should be elicited. We aimed to compare health state utility estimates for PTMC as derived from general population volunteers (GenPop) and thyroid cancer survivors (TCSurv).

**Methods:**

GenPop and TCSurv completed a time trade-off task for 10 PTMC health states described by clinical vignettes. Health utilities were compared between groups with univariate and multivariable linear regression, adjusting for age, sex, and income. Subgroup analysis was performed for health states with and without treatment complications.

**Results:**

70 GenPop and 72 TCSurv completed the surveys. GenPop reported lower utilities relative to TCSurv for all 10 health states, with an effect size of 0.044 attributed to participant group in the multivariable analysis (*p* = 0.01). This observation persisted in stratified analysis by treatment complication, with effect sizes 0.047 (*p* = 0.04) and 0.042 (*p* < 0.01) for uncomplicated and complicated groups, respectively. Health utilities were lower for complicated scenarios (effect size 0.067, *p* < 0.001) compared to uncomplicated scenarios.

**Conclusion:**

For 10 low-risk thyroid cancer health states, GenPop reported significantly lower health utilities than TCSurv. Health economists and healthcare delivery scientists should be aware of these differences when integrating health utilities into comparative-effectiveness research.

**Supplementary Information:**

The online version contains supplementary material available at 10.1007/s11136-025-04033-7.

## Abstract (plain language summary)

Small, low-risk thyroid cancers have excellent survival rates, regardless of which treatment is chosen. Options include surgical removal of the thyroid, burning and killing the cancer with a needle inserted through the skin, and leaving the cancer in place and monitoring it with imaging. Understanding preferences for these treatment options is informative both to clinicians and to health care policy researchers. To quantify these preferences, we asked thyroid cancer survivors and general public volunteers to rate the quality of life of 10 hypothetical thyroid cancer treatment scenarios. To determine this rating, we asked: if you had a certain health problem and only had 10 years left to live, how much time would you give up out of those 10 years to live the rest of your life without the health problem? We found that for all scenarios, the general public volunteers were willing to give up more time (approximately 5 months out of the 10 years) than the cancer survivors. This finding suggests the general public perceived the quality of life in these thyroid cancer treatment scenarios to be lower than the cancer survivors did. Characteristics like age, income, race, sex, education level, and health insurance type did not affect this difference between groups. Since researchers use these value assessments when comparing the cost-effectiveness of treatments for low-risk thyroid cancer, they should be aware that the assessments may differ by the population that provides them.

**Supplementary Information:**

The online version contains supplementary material available at 10.1007/s11136-025-04033-7.

## Background

Small (≤ 1 cm), well-differentiated papillary thyroid cancers are collectively termed papillary thyroid microcarcinomas (PTMC) and are characterized by slow growth and excellent prognosis [1]. Over the last 10 years, management strategies have expanded from surgical resection to include less invasive options like radiofrequency ablation and active surveillance [2, 3]. This shift emphasizes the importance of shared decision-making to choose the right treatment for each patient.

There is a prevailing impression among healthcare practitioners that patients prefer surgical resection for these low-risk cancers, however, whether this preference holds true in reality is unknown [4]. The gold standard measurement of health-related quality of life in health economics is health utility multiplied by time, expressed in quality adjusted life years (QALYs) [5]. Health utility is measured on a scale of 0 to 1, where 0 = death and 1 = perfect health. For thyroid cancer, health state utilities have historically been derived from quality-of-life instruments. These instruments have highly variable results and proven susceptibility to ceiling effect [6, 7].

Conventionally, health utility estimates are acquired from cohorts of general population volunteers, a practice that is founded in the ethical theory that, compared to patients, these individuals are less susceptible to bias and better represent taxpayers who ultimately contribute to payment of medical care [8–10]. Additionally, “generic” estimates are helpful to compare between diseases. However, the derivation of health utilities from patients has been increasingly explored [11, 12]. Some advocate that the aforementioned “bias” that patients introduce into value assessments is not problematic but useful, as they bring a realistic perspective informed by their lived experience with disease [13]. Indeed, studies in breast cancer have shown that general population health utilities are lower relative to breast cancer survivors [14]. This finding has not been studied in thyroid cancer. Establishing this difference is important because utility estimates ultimately drive comparative- and cost-effectiveness studies that carry implications for societal resource allocation.

The purpose of this study was to compare health state utility estimates for low-risk papillary thyroid cancer as derived from general population volunteers and thyroid cancer survivors. We hypothesized that, similar to data from breast cancer, the general population would report lower health state utilities compared to thyroid cancer survivors.

## Methods

### Participant selection

We conducted a cross-sectional, proctored survey of general population and thyroid cancer survivors. General population participants were recruited through ResearchMatch.org. ResearchMatch.org is a national electronic, web-based recruitment tool that was created through the NIH Clinical & Translational Science Awards Consortium as an Institutional Review Board (IRB)-approved data repository. Individuals with a personal history of thyroid disease or thyroid cancer were excluded from the general population cohort.

Thyroid cancer survivors were recruited from two sources. First, patients treated for early-stage thyroid cancer were identified retrospectively from the University of Maryland’s tumor registry and electronic medical records spanning 1/1/2015 through 12/31/2022. Second, thyroid cancer survivors were also recruited from ResearchMatch.org. Inclusion criteria was personal history of thyroid cancer defined as one of the following: thyroid cancer diagnosed by fine-needle aspiration (FNA) or surgical specimen, thyroid mass suspicious for thyroid cancer based on FNA (Bethesda III-V), or thyroid mass determined to be probably malignant (TI-RADS 5) on ultrasound. Additional inclusion criteria for both groups included age *≥* 18 years and English fluency; individuals were excluded if they lacked the mental capacity to answer study questions.

Verbal informed consent was obtained from each participant at the start of each study session as approved by the IRB. Participants received renumeration of $30 for their participation. All recruitment, enrollment, and data collection occurred between August 2022 and March 2024. The study protocol was reviewed by the University of Maryland IRB and determined to be exempt (HM-HP-00093873-11).

### Sample size calculation

To determine the sample size, we enrolled a pilot cohort of participants (*n* = 10 / cohort) and calculated the difference in means for each health state. We identified the smallest difference between groups and calculated the pooled standard deviation of mean differences across all 10 health state comparisons, then used these to conduct a power analysis. To detect this difference with 80% power and 5% type 1 error rate, we estimated 94 participants per group. An interim analysis was completed once accrual reached 70 per cohort, which demonstrated that the primary endpoint—a significant interaction between participant type and mean utility—was already met. Thus, this phase of the study was terminated early and submitted for analysis. Additional accrual of thyroid cancer survivors continued beyond this analysis, the full utility results of which are published in a separate article [15].

### Study variables

The dependent variable was utility, a continuous variable with values from zero to one whose definition is described below. We used an optional demographics survey to collect demographic variables for all participants, including age, sex, income, race, ethnicity, education level, employment status, marital status, insurance type, and residence type. Of these, only the first three were included as independent predictors for analysis; the remaining were used descriptively to characterize our study cohort. Additionally, we collected clinical data from our cancer survivor participants to assess the generalizability of our sample to the population of thyroid cancer survivors; these variables included tumor size, lymph node status, procedure type, tumor pathology, and treatment-related complications. Treatment-related complications were not mutually exclusive and were defined as:

- Hypoparathyroidism: (1) low parathyroid hormone (PTH) level postoperatively, or (2) requirement of intravenous calcium with prolonged hospital stay *≥* 2 nights. Permanent hypoparathyroidism was defined as: (1) need for calcium supplementation or (2) persistently low PTH for ≥ 6 months.
- Recurrent laryngeal nerve damage: (1) vocal cord immobility as evidenced by laryngoscopy, or (2) vocal cord augmentation injection. Permanent nerve damage was defined as injury persisting ≥ 6 months.
- Hematoma (i.e. bleeding) requiring intervention and other complications requiring intervention were also included.

This clinical data was obtained through retrospective chart review and thus was only available for participants recruited from the hospital registry (not from ResearchMatch.org).

### Utility estimation

To estimate utilities, participants underwent a structured proctored interview to complete a time trade-off (TTO) task. The TTO task consisted of TTO valuations for 10 PTMC health states including 4 uncomplicated treatment scenarios (active surveillance, radiofrequency ablation, thyroid lobectomy, total thyroidectomy) and 6 complicated treatment scenarios (temporary and permanent nerve injury, hypocalcemia, cancer progression). The collaborative development of these health state descriptions is described in a prior study [16].

The survey was administered to individual participants via video conference sessions. The facilitator and the participant first completed a tutorial of the TTO task, which utilized a scenario about an unrelated health condition (hearing loss). Then, they sequentially reviewed the 10 PTMC health state vignettes in an order randomized by treatment type. For each vignette, the participant would first read one of 10 thyroid cancer health states and a perfect health state comparator. The participant was then prompted to complete a standard TTO task to discern the amount of time in perfect health the participant values as equal to living 10 years in a given PTMC health state; an example is provided in Online Resource 1. To elicit this, participants were given 3 possible answer choices to start with: (1) I choose to live 10 years with the described thyroid cancer health state, or (2) I choose to 10 years with perfect health, or (3) I value these options equally. The participant was then presented with subsequent choices of different amounts of time in perfect health until equivalency between 10 years in the thyroid cancer health state and an amount of time in perfect health was met. The lengths of time in perfect health presented ranged from a minimum of 5 years to a maximum of 10 years, with 2-month trading increments. Alternatively, participants could opt to manually enter the amount of time in perfect health that they consider to be equal to 10 years of the thyroid cancer health state, e.g. “9 years and 4 months”. With this feature, participants could manually enter less than 5 years.

### Analysis

The dependent variable was utility. Independent predictors, with the indicated (*) reference group, included participant group (thyroid cancer survivors vs. general population*), presence of complications in the associated vignette (complicated vs. non-complicated*), and participant age (continuous, per year), sex (female vs. male*), and income (<$75,000* vs. ≥ $75,000 per year).

We compared baseline characteristics between the two participant groups with descriptive statistics; Chi-squared test or Fisher’s exact test were employed as appropriate for categorical variables, and Student’s t-test for continuous variables. The independent predictors were assessed for association with the dependent outcome (health utility) through univariate and multivariable linear regression. Variables with anticipated clinical relevancy or with an association at the *p* < 0.1 level on univariate analyses were included in multivariable regression.

Descriptive statistics demonstrated clustering of utilities data among uncomplicated health states, while complicated health states yielded more variable utility results, raising suspicion for health state treatment complications as a potential effect modifier. Thus, we performed a sensitivity analysis by stratifying the multivariable regression by the presence of complications in the health states.

Data missingness was overall rare. No participants lacked outcome data. Among the demographic variables, data missingness occurred at a rate of 0.7% (*n* = 1) for education, employment status, insurance status, and community type; 2% (*n* = 3) race; and 6% (*n* = 9, split 6 general population and 3 cancer survivors) for income. Clinical data was limited to the 67% who were recruited through the hospital registry; of these, only 1 participant had missing data (for tumor size only). A complete-case analysis approach was used for regression, which excluded only the 9 participants with missing income data.

## Results

### Baseline characteristics

For general population recruitment through ResearchMatch, 471 individuals were contacted and 70 (15%) agreed to participate, 1 of which had a history of thyroid cancer and enrolled as a thyroid cancer survivor participant. For thyroid cancer survivor recruitment through ResearchMatch, 56 individuals were contacted and 23 agreed to participate (41%), 1 of which did not have a history of thyroid cancer and enrolled as a general population participant. The remaining thyroid cancer survivor participants were recruited from the hospital electronic medical records; 439 met clinical search criteria, 4 were deceased and 82 did not have listed or working email addresses. Thus, 357 potential participants were contacted and 49 (14%) agreed to participate, bringing the thyroid cancer survivor total to 72. Thus, in total, we accrued 70 general population volunteers and 72 thyroid cancer survivors to participate in the study, all of whom were included in analysis.

Participant characteristics are presented in Table 1. The general population participants differed from thyroid cancer survivors by age (39 vs. 55 years, *p* < 0.001), highest level of education attained (29% vs. 13% high school, *p* = 0.011), marital status (51% vs. 68% married, *p* = 0.004), annual household income (43% vs. 26% <$75,000, *p* < 0.001), and residence type (36% vs. 21% urban, *p* = 0.032). There were no statistical differences between groups for gender, race, ethnicity, employment status, or insurance type.

Of the 72 thyroid cancer survivors, 24 (33%) did not have retrospective clinical data available. Of those with available clinical data, the average cancer size was 1.5 cm, 51% were node positive, all demonstrated papillary thyroid carcinoma on pathology, and most (83%) underwent total thyroidectomy, of which more than half (56%) included a lymph node dissection or node excision. Most (94%) cancer survivors received thyroid hormone replacement after surgery. Treatment-related complications included vocal cord palsy (10%), hypocalcemia (6%), and bleeding (4%). Of the 5 participants who had “other” complications, this included a wound infection requiring antibiotics (*n* = 1), a deep vein thrombosis (*n* = 1), pleural injury during dissection requiring chest tube (*n* = 1), chyle leak requiring admission, diet modification, and needle-based aspiration (*n* = 1), and esophageal and tracheal injury requiring tracheostomy and gastrostomy tube placement (*n* = 1).

**Table 1:** Baseline characteristics of cohorts

	Overall	Thyroid Cancer Survivors	General Population	*p*-value
	N = 142	N = 72	N = 70	
Age	49 (34–65)	55 (45–66)	39 (29–60)	< 0.001
Sex: Female	102 (72%)	49 (68%)	53 (76%)	0.119
Race				0.165
White or Caucasian	118 (83%)	61 (85%)	57 (81%)	
Black or African-American	10 (7%)	2 (3%)	8 (11%)	
Asian	7 (5%)	5 (7%)	2 (3%)	
Other or prefer not to say	7 (5%)	4 (6%)	3 (4%)	
Ethnicity: Non-Hispanic or Latino	138 (97%)	72 (100%)	66 (94%)	0.057
Highest level of education completed				0.01
High school	29 (21%)	9 (13%)	20 (29%)	
College	50 (35%)	27 (38%)	23 (33%)	
Post-graduate	62 (44%)	35 (49%)	27 (38%)	
Marital status: Married	85 (60%)	49 (68%)	36 (51%)	0.004
Employment status: Employed	97 (69%)	49 (69%)	48 (69%)	0.885
Household income: $75,000 and above	86 (65%)	51 (74%)	35 (55%)	< 0.001
Insurance type: Private insurance	107 (76%)	51 (72%)	56 (80%)	0.082
Type of residence				0.032
Urban	40 (28%)	15 (21%)	25 (36%)	
Suburban	82 (58%)	46 (65%)	36 (51%)	
Rural	19 (14%)	10 (14%)	9 (13%)	

Continuous variables reported as median (q1,q3). Categorical variables reported as count (percentage)

### Primary outcome

**Fig. 1:**
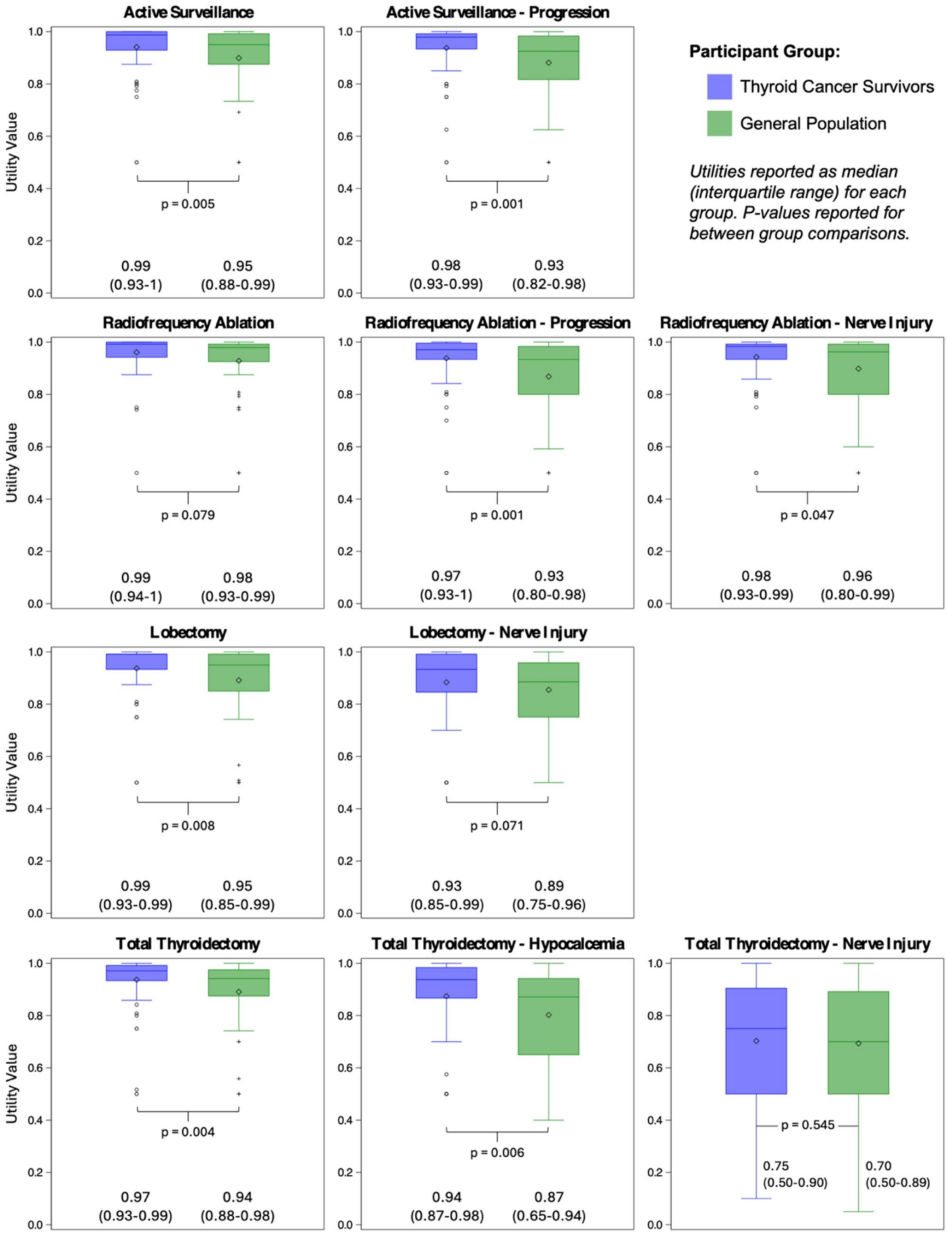
Health State Utilities for 10 Papillary Thyroid Microcarcinoma Health States, Elicited from General Population and Thyroid Cancer Survivors

1,420 utilities were estimated. General population volunteers reported lower utilities relative to thyroid cancer survivors for all 10 health states, as reported Fig. 1. Univariate analyses demonstrated that complicated vignettes (relative to uncomplicated) and participant group (thyroid cancer survivor vs. general population) impacted utility assignments (effect sizes − 0.067 with *p* < 0.001, and 0.045 with *p* = 0.011, respectively). These effects were persistent on multivariable analysis.

Income, vignette complications, and participant group met the a priori criteria for inclusion in the multivariable analysis; age and sex were not associated with utility on univariate analysis, but were felt by the investigators to be clinically relevant and thus were also included in the adjusted model. In the multivariable analysis, the effect size attributed to participant group was 0.044 (95% Confidence Interval [C.I.] 0.01–0.08, *p* = 0.014). In the TTO analysis, this effect size can be thought of as how much more time in a ten-year lifespan the general population participants would be willing to “give up” to live with perfect health instead of thyroid cancer, compared to the corresponding responses from thyroid cancer survivors. This effect size translates to 0.044 × 120 months = 5.28 months. Univariate and multivariable analyses are shown in Table 2.

**Table 2:** Effect on utility, univariate and multivariable analyses

	Univariate Analysis		Multivariable Analysis	
	Effect (95% Confidence Interval)	*p*-value	Effect (95% Confidence Interval)	*p*-value
Age (continuous, per 1 year)	0 (-0.001, 0.001)	0.872	-0.001 (-0.002, 0)	0.162
Sex (vs. male)	0.016 (-0.02, 0.06)	0.411	0.008 (-0.03, 0.05)	0.671
Income (vs. < $75K)	0.033 (0, 0.07)	0.068	0.028 (-0.01, 0.06)	0.124
Complicated vignettes (vs. non-complicated)	-0.067 (-0.08, -0.05)	< 0.001	-0.066 (-0.08, -0.05)	< 0.001
Thyroid Cancer Survivors (vs. General Population)	0.045 (0.01, 0.08)	0.011	0.044 (0.01, 0.08)	0.014

### Stratified analysis

In the stratified sensitivity analysis of vignettes with and without complications, general population participants still reported lower utility relative to thyroid cancer survivors in both subgroups (effect size 0.042 with *p* = 0.041 for complicated, and 0.047 *p* = 0.006 for uncomplicated). These findings are shown in Table 3. The effect of age on utility assignment was statistically significant for the multivariable analysis of the uncomplicated scenarios only, but this effect size was quite small (-0.001, 95% C.I. -0.002 to 0, *p* = 0.028) and likely not clinically meaningful given its isolated association in this subgroup.

**Table 3:** Effect on utility, stratified multivariable analysis by treatment complication

	Complicated Vignettes		Non-complicated Vignettes	
	Effect (95% Confidence Interval)	*p*-value	Effect (95% Confidence Interval)	*p*-value
Age (continuous, per 1 year)	-0.001 (-0.002, 0.001)	0.417	-0.001 (-0.002, 0)	0.028
Sex (vs. male)	0.007 (-0.04, 0.05)	0.747	0.010 (-0.03,0.04)	0.593
Income (vs. < $75K)	0.032 (-0.04, 0.05)	0.118	0.021 (-0.01, 0.05)	0.221
Thyroid Cancer Survivors (vs. General Population)	0.042 (0, 0.08)	0.041	0.047 (0.01, 0.08)	0.006

## Discussion

To our knowledge, this is the first study to compare preference-based health utilities between the general population and thyroid cancer survivors, and the largest to use gold-standard methodology (TTO) to elicit preference-based health utilities within thyroid cancer. The use of TTO in this study is critical, as prior research in thyroid cancer has shown that traditional quality-of-life instruments such as the EQ-5D, SF-6D, and HUI are non-responsive in this condition and highly susceptible to ceiling effect [6]. This may in part be due to the fact that treatments for early-stage thyroid cancer are well-tolerated by most patients. Therefore, traditional components of health utility indexes—pain, fatigue, nausea, etc.—are common in many diseases but less relevant in thyroid cancer. Our data suggest that the general population consistently assigns lower utilities to small, low-risk thyroid cancer health states compared to thyroid cancer survivors, which is consistent with our study hypothesis and echoes similar findings from breast cancer studies. Additionally, as expected, complicated scenarios were assigned lower health utility scores compared to uncomplicated health states.

Differences between health utility assignments by the general population and by patients appear to vary across diseases. In one study using TTO, compared to colorectal and breast cancer patients, the general population assigned higher health utilities to the same SF-6Dv2-defined general health states, and this difference widened with poorer health states [17]. In contrast, a large meta-analysis in 2010 compared 30 studies reporting TTO data for various health states collected from patients and the general population. They found that there was no difference between health state utilities when TTO was used with hypothetical vignettes, but that patients assigned higher health utilities than the general population when the TTO was used with their own health state [18]. Conversely, Kaur et al. reported that general population volunteers undervalue breast cancer health states compared to cancer survivors [14]. Using a standard gamble method for prostate cancer health states, Gries et al. similarly reported a trend that the general public assigned lower health utilities to compared to cancer survivors [19]. Health economists should be aware of these established differences in valuations between patients and the general population when selecting a sample for the derivation of health utilities.

There has long been a debate about whose preferences ought to be utilized for cost-effectiveness analysis. Advocates of the utilization of public preferences emphasize the importance of capturing the societal perspective for resource allocation, the downplaying of impairment severity by patients due to their adaptation to it, and the insurance principle (e.g. individual, insured patients will not bear the cost of care and thus may be more willing to accept expensive, minimal gain therapies than is justified by the associated societal financial burden) [13, 20]. Advocates of the utilization of patient preferences believe the patients’ perspectives of their own lived experience is more informative and accurate than the public’s hypothetical perspective, citing the disability bias phenomenon (e.g. individuals with lived experience with a chronic illness or disability often rate their quality of life higher than those who have not experienced that health state) [13, 20, 21]. Others advocate that both public and patient preferences should be considered, either by creating separate cost effectiveness models or by providing the public with patient-derived preferences during their valuations [20, 22]. The consideration of both valuations may be ideal, but it may not always be practical to obtain preferences from more than one population. This debate has not reached a consensus; our team believes that health utilities should ideally be derived from samples of the population to which they are being applied, and that utilities from the general population should be utilized in health economics models with caution. Importantly, relative advantages of each study population may also vary depending on the disease being studied.

There are several limitations to this study. First, both of our cohorts are subject to sampling bias, which may limit the validity of the derived utilities. On average, our thyroid cancer survivor participants were older, had more advanced disease, and underwent more extensive surgery than is typical of patients with PTMC [23, 24]. Accruing an adequately powered PTMC-exclusive survivor cohort would require a multi-institutional collaborative network. Nevertheless, given the excellent prognosis of non-metastatic thyroid cancer and favorable quality of life among treated patients, we believe that our data should approximate those of a PTMC cohort [25–27]. Regarding the general population, our sample approximated 2020 census data for age (median: 39 years, though this does not exclude children), income (national median income: $78,719), marital status (51% in our sample married vs. 47% nationally), employment status (69% vs. 61% employed), living in non-rural environments (87% vs. 80%), and White race (81% vs. 75%) [28]. Some features were over-represented such as college degree (71% vs. 35%) and non-Hispanic ethnicity (94% vs. 80%) [28]. This comparison suggests that our sample may not be fully generalizable, though the direction of the potential bias this may have introduced to our utility data is uncertain. Both the general population and cancer survivor groups had substantial non-response rates, which likely contributes to the limited generalizability noted above. Relatedly, there were demographic differences between our study’s participant cohorts. Prior research suggests that higher socioeconomic status (as is the case in our cancer survivor sample) is associated with higher health utility valuations, and that older age (as is the case in our cancer survivor sample) may be associated with lower health utility values [29, 30]. We adjusted for age and income in our multivariable regression model and neither were significantly associated with reported utility. Third, we designed the TTO task based on previously published thyroid cancer utility values, which are predominantly > 0.8 [7]. Therefore, we designed our task with a range of 0.5 to 1.0 to reduce survey fatigue. The potential floor effect of this approach was mitigated by participants’ ability to manually enter any number between 0 and 10 years. Still, this approach may have biased our utilities towards higher values. Fourth, our study vignettes, though well-validated, by nature cannot encompass all aspects of each health state [16, 31]. Examples of missing information include out-of-pocket costs and adverse effects of potential future treatments. Finally, the TTO technique itself has inherent limitations, such as a more pronounced ceiling effect among participants who are parents, the confounding effect of participants whose own life expectancy is less than 10 years, difficulties of cognitive understanding the trade-off task, and interviewer effect [32–34]. We mitigated the latter two by (a) providing a tutorial and a proctor, and (b) by standardizing training across proctors.

Acknowledging these limitations, this study aligns with prior literature that suggests health utilities may vary by the population from which they are derived. This phenomenon has now been demonstrated for breast cancer and low-risk thyroid cancer health states; future research could apply a similar approach to other disease states such as more advanced thyroid cancers, other malignancies, and benign diseases. A mixed-methods investigation of the drivers of the difference in utility between the general population and cancer survivors could also elucidate which cancer health states may be appropriately approximated by data from a general population sample. Health services researchers should consider the source of utilities integrated into the cost- and comparative-effectiveness models they create and evaluate, as models may be sensitive to the utility differences incurred by utilizing different derivation populations.

## Conclusion

For 10 low-risk thyroid cancer health states, general population participants reported significantly lower health utility values than thyroid cancer survivors when adjusting for age, sex, and income. These differences should be considered when integrating health utilities into comparative-effectiveness research.

## Electronic supplementary material

Below is the link to the electronic supplementary material.

**Supplementary Material 1**: **Online resource 1**: This supplemental information provides an example of a time trade-off scenario with a clinical vignette.
