# Supplementary material for: Differences in health state valuation for small, low-risk thyroid cancer between general population and cancer survivors: a cross-sectional analysis
Source: Qual Life Res. 2025 Aug 23;34(10):2891–900. doi: 10.1007/s11136-025-04033-7 (PMC12535529; doi:10.1007/s11136-025-04033-7)
Supplement: Supplementary file 1 — Supplementary Material 1: Online resource 1: This supplemental information provides an example of a time trade-off scenario with a clinical vignette. [file 11136_2025_4033_MOESM1_ESM.docx]

**Title:** “Differences in health state valuation for small, low-risk thyroid cancer between general population and cancer survivors: A Cross-Sectional Analysis”

**Journal:** Quality of Life Research

**Authors:** Kendyl Carlisle, Rebecca Kowalski, Aprill N. Park, Salome Ricci, Kai Sun, Carrie Cunningham, Julia F. Slejko, C. Daniel Mullins, Yinin Hu

**Corresponding Author**: Yinin Hu, MD, University of Maryland School of Medicine, Department of Surgery, Baltimore, MD, USA, E: [yinin.hu@som.umaryland.edu](mailto:yinin.hu@som.umaryland.edu)

**Online Resource 1.** Example of a Time Trade-Off Scenario with Clinical Vignette

Here, we describe an example of a time trade-off scenario including a clinical vignette. Each scenario begins with the instructional text: “Please carefully read the two health scenarios below (Option A, Option B). One scenario may seem more desirable than the other. Imagine that you will live in one of the two conditions for several years, and then die. Which option would you prefer?”

This is followed by a clinical vignette and subsequent answer choices (Fig 1). The clinical vignette includes a description of the cancer diagnosis and treatment, symptoms, additional treatment required, impact on daily life, and other notes on quality of life; these 10 vignettes have been previously published elsewhere [1]. Regarding the answer choices, if the participant selects “I choose to answer manually,” the participant is directed to input the number of years & months living in Option B that they prefer equally to living for 10 years in Option A (e.g. “9 years and 6 months”).

**
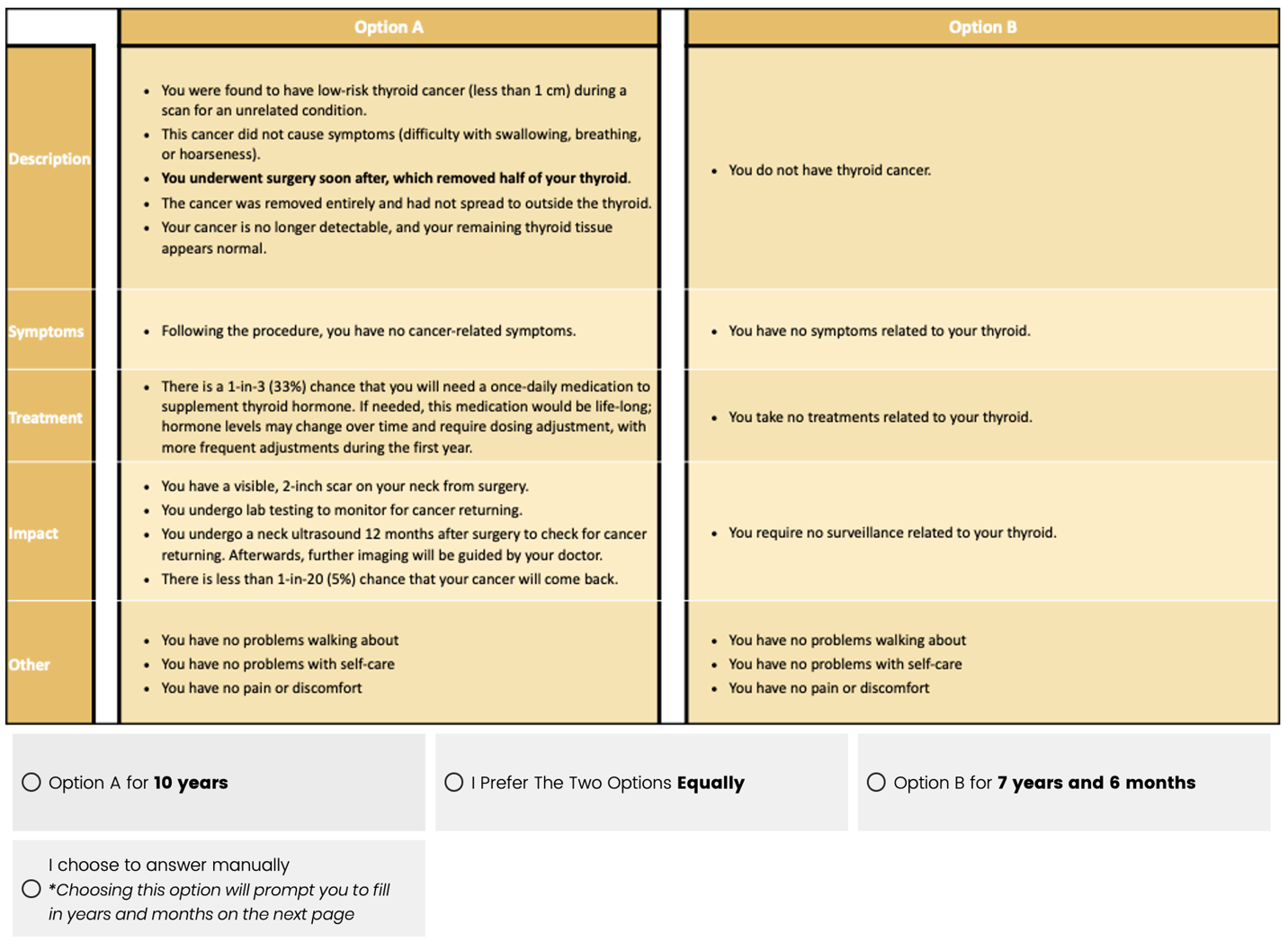
**

**Fig 1:** Example Clinical Vignette Used in Time Trade-Off Survey Instrument

**References**:

1. Roth, E. G., Kim, J., Slejko, J. F., Mullins, C. D., Doyle, J. L., Levitt, D. L., Melendez, M., Fletke, K. J., & Hu, Y. (2023). Constructing Health State Descriptions for Low-Risk Thyroid Cancer: Stakeholder Engagement and Formative Qualitative Research. *The patient*, *16*(1), 67–76. https://doi.org/10.1007/s40271-022-00597-5
